# Oral health and orofacial pain in people with dementia admitted to acute hospital wards: observational cohort study

**DOI:** 10.1186/s12877-018-0810-7

**Published:** 2018-05-23

**Authors:** Liza J. M. van de Rijt, Roxane A. F. Weijenberg, Alexandra R. Feast, Victoria Vickerstaff, Frank Lobbezoo, Elizabeth L. Sampson

**Affiliations:** 1 Faculty of Dentistry, Department of Oral Kinesiology, Academic Centre of Dentistry Amsterdam (ACTA), University of Amsterdam and Vrije Universiteit Amsterdam, Amsterdam, The Netherlands; 2 Marie Curie Palliative Care Research Department, Division of Psychiatry, University College London, London, UK; 3 The Research Department of Primary Care and Population Health, University College London, London, UK; 4 Barnet Enfield and Haringey Mental Health Trust Liaison Psychiatry Team, North Middlesex University Hospital, London, UK

**Keywords:** Dementia, Orofacial pain, Acute hospital, Oral health, OPS-NVI

## Abstract

**Background:**

Orofacial pain in people with dementia is difficult to detect, and often under-treated. Our aim was to investigate the prevalence of orofacial pain in people with dementia in acute hospitals in the UK. Secondary aims were to examine oral health status and explore associations between orofacial pain and oral health factors.

**Methods:**

This cross-sectional observational study was carried out in two UK hospitals. Using the Orofacial Pain Scale in Non-Verbal Individuals (OPS-NVI) to identify orofacial pain, 101 participants with dementia, admitted to acute medical wards, were observed for at least 3 min during rest and chewing. Verbal participants were then asked about presence of orofacial pain, using self-report pain scales. Finally, a brief oral assessment was performed.

**Results:**

Orofacial pain, assessed with the OPS-NVI, was present in 11.9% (95% C.I. 5.9, 18.8) of participants at rest and 21.9% (95% C.I. 14.6, 31.3) whilst chewing. Participants who were no longer able to self-report pain were significantly more likely to experience orofacial pain. Oral health in both dentate and edentate participants was poor. Brush frequency, indication of chewing quality, consistency of the food, presence of extra-oral abnormalities, person who performed mouth care, and oral hygiene in dentate participants were significant predictors for the presence of orofacial pain.

**Conclusion:**

Improving oral care in acute hospital patients with dementia, particularly those who cannot self-report pain, may significantly reduce pain and suffering in this population.

**Electronic supplementary material:**

The online version of this article (10.1186/s12877-018-0810-7) contains supplementary material, which is available to authorized users.

## Background

Due to global ageing, the prevalence of dementia will double every 20 years, with an expected 115 million people with dementia by 2050 [1]. Approximately 50% of people with dementia experience pain daily [2]. This can be difficult to detect, and is therefore often under-treated [3].

Orofacial pain is common in people aged 70 years or above and may be caused by teeth or their supporting tissues, the muscles and joints of the masticatory system, or other non-odontogenic tissues [4, 5]. In the general population, oral health problems increase with age [6]. In people with dementia, oral health problems are even more common; they might develop apraxia and become unable to care for their mouth and teeth, or in the more advanced stages, they may resist care [7]. Conversely, poor oral health may be a risk factor for the development of cognitive impairment, associated with malnutrition or diminished stress regulation ability [8].

Oral health problems are one of the main causes of orofacial pain [9]. The prevalence of orofacial pain in older people aged 60 years or above without dementia is 6.7–18.5% and the few studies reporting on orofacial pain in people aged 60 years or above with dementia give a prevalence between 7.4 and 21.7% [9–11].

For pain assessment, self-report pain scales are the ‘gold standard’, but it is vital that the person is able to understand what the task involves and is able to communicate how they rate themselves on the scales [12]. Some people with dementia may be unable to answer simple ‘yes or no’ questions, and therefore self-reported pain assessment is not suitable, and direct observation is needed [2, 12]. The Orofacial-Pain Scale for Non-Verbal Individuals (OPS-NVI), is currently being developed to diagnose orofacial pain in patients who are unable to communicate verbally [2].

In the UK, annually, 25% of people with dementia have an admission to an acute hospital and there is often no routine assessment of pain [13]. Older people admitted to acute hospitals often have poor oral health and the risk of this is increased for people with dementia [14].

### Aims

The primary aim of this study was to examine the prevalence of orofacial pain in people older than 70 years with dementia admitted to UK acute hospitals. The secondary aims were to examine oral health of people with dementia admitted to acute hospital and to explore associations between orofacial pain and oral health factors.

## Methods

### Study design and participants

Data were collected cross-sectionally by one researcher, on older people’s wards of two hospitals. Hospital 1 is located in central London, hospital 2 is located in the suburbs of London. Participants were eligible for inclusion if they were aged 70 years or above, had a clinical diagnosis of dementia, and their English language was sufficient to complete the study ratings. Patients who indicated verbally, or non-verbally, that they did not wish to participate, those with delirium, or those with clinical concerns were also excluded.

### Ethics approval and consent

The procedure for obtaining informed consent was developed to comply with capacity legislation governing England and Wales (Mental Capacity Act 2005, Sections 30–34). Informed consent was obtained from participants with the capacity to consent. If they did not have capacity, a personal or professional consultee was asked to give agreement for the person’s participation, and sign his/her agreement for this. The study was reviewed and approved by the London Queen Square Research Ethical Committee (17/LO/0430) and the UK Health Research Authority.

### Measurement instruments

Demographic information was collected on age, gender, ethnicity, marital status, number of years schooling in general education, and highest completed level of education. The components ‘resting’ and ‘chewing’ of the OPS-NVI were used to identify orofacial pain [2]. During a single assessment, the participant was observed for 3 min during rest, and for 3 min during eating a routine meal. For each activity a score sheet of the OPS-NVI was completed during, or immediately after the observation. Behaviour items of the categories ‘facial activities’, ‘body movements’, ‘vocalizations’, and ‘specific’ were scored ‘yes’, ‘no’, or ‘not applicable’. These items are shown in Table 1.

**Table 1:** Behaviour items of the OPS-NVI

Category	Behaviour
Facial activities	Frowning Narrowing or closing eyes Raising upper lip Opened mouth Tightened lips
Body movements	Resisting care Guarding Rubbing Restlessness
Vocalizations	Using offensive words Using pain-related words Screaming/shouting Groaning
Specific	Restricting jaw movement Refusing prosthetics Drooling

*OPS-NVI* Orofacial-Pain Scale for Non-Verbal Individuals

For each activity, the researcher estimated the perceived pain intensity on a scale between 0 and 10, where 0 is no pain and 10 is as bad as it possibly could be [15]. For participants who were able to communicate verbally, brief self-report pain scales, the Numeric Rating Scale (NRS), the Verbal Descriptor Scale (VDS), and the Faces Pain Scale Revised (FPS-R), were used to identify orofacial pain during activity [16]. To determine whether the participants could self-report, their understanding of the scales was assessed. Test-questions were used to determine whether they understood the scales. For example, the participants were asked ‘Which number reflects more pain; a 2 or an 8?’

Prescribed medication was documented; analgesics, antidepressants, antiepileptics, and/or antipsychotics. A brief oral assessment was performed to evaluate multiple oral health factors: Information was collected on last visit to a dentist, usual brush frequency prior to admission, indication of quality of swallowing and chewing, consistency of food, and mouth care. If the participant was no longer able to provide this information, a family carer or nurse was asked.

During extra-oral examination, the face of the participant was observed. If present, extra-oral abnormalities, for example wounds or bumps, were documented. If present, participants’ dentures were examined for retention (how well the denture is fixated in the mouth), occlusion (the contact between upper and lower denture), vertical dimension (when upper and lower denture are in contact), and hygiene. Denture hygiene was examined by dissolving five Dental Plaque Disclosing Tablets (Mira-2-Ton, Hager Werken, Duisburg, Germany) in water, and placing the denture in the water for 30 s. Thereafter, the denture was rinsed, and the Denture Hygiene Index (DHI) was recorded, as ‘excellent’, ‘fair’, or ‘poor’ [17]. For dentate participants, participants with remaining natural teeth, the number of present teeth, missing teeth, and retained roots were counted. As a proxy for chewing ability, the number of pairs of opposing lower and upper teeth, occlusal units (OU), were counted [18]. The oral hygiene of the dentate participants was examined, using the Debris Index (DI) of the Simplified Oral Hygiene Index (OHI-S), with a range from 0.0 to 3.0 [19].

### Sample size

The prevalence of orofacial pain in older people without dementia was 6.7–18.5% and in older people with dementia 7.4–21.7% [9–11]. Based on these results and taking the cautious approach, a prevalence of 12% was assumed to estimate the sample size. Aiming at a precision of +/− 5 percentage points, with a level of confidence of 95%, 162 participants were needed [20]. See Additional file 1 for the sample size calculation.

### Data analysis

SPSS Version 24 Software (IBM Corp., Armonk, NY, USA, 2012) was used for analyses. Participant characteristics and oral health factors were described by means, standard deviations, ranges, and percentages. The prevalence of orofacial pain was reported with 95% confidence intervals. During the observation with the OPS-NVI, pain intensity was estimated by the researcher on a scale of 0 to 10. For the presence of pain, outcomes were analysed as ‘yes, pain is present’, when pain intensity was rated greater than, or equal to 1. The outcomes were analysed as ‘no, pain is not present’, when pain intensity was rated 0. For the participants who were able to complete the self-report pain scales correctly, the prevalence of orofacial pain, using these scales, was reported. To determine whether an oral health factor was a predictor variable for the presence of orofacial pain, according to the OPS-NVI (response variable), the odds ratio, with the corresponding confidence interval, were calculated by performing binary logistic regression. The odds ratio represents the odds that orofacial pain will occur given the presence of a particular oral health factor, compared to the odds that orofacial pain will occur given the absence of a particular oral health factor.

## Results

**Fig. 1:**
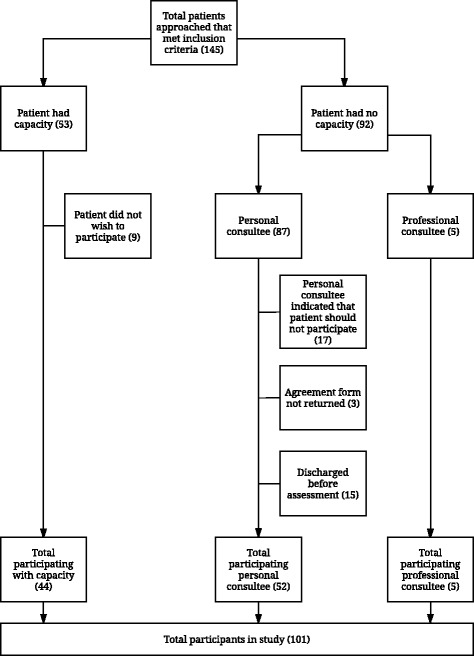
Recruitment flowchart

In total, 145 patients that met the inclusion criteria were approached and 101 patients were recruited. Of the 44 patients that were approached, but not included in the study, 9 were excluded because they did not wish to participate, 17 were excluded because the personal consultee indicated that the patient should not participate, 3 were excluded because the personal consultee, who gave verbal agreement, did not return the signed consultee form, and 15 were excluded because they were discharged from hospital before they could be screened (See Fig. 1). The average age was 85.6 (SD 6.68) years old, and 69.3% were female. Further demographic features are given in Table 2.

**Table 2:** Descriptive analysis of demographic characteristics of all participants and of each hospital separately

	Total (*n* = 101)	Hospital 1 (*n* = 22)	Hospital 2 (*n* = 79)
Gender [n (%)]			
Female	70 (69.3)	12 (54.5)	58 (73.4)
Male	31 (30.7)	10 (45.5)	21 (26.6)
Age *M, SD* (range)	85.6, 6.68 (70–99)	84.1, 7.02 (70–98)	86.0, 6.56 (72–99)
Ethnicity [n (%)]			
White	70 (69.3)	19 (86.4)	51 (64.6)
Mixed/Multiple ethnic groups	0 (0)	0 (0)	0 (0)
Asian/Asian British	8 (7.9)	1 (4.5)	7 (8.9)
Black/African/Caribbean/Black British	11 (10.9)	1 (4.5)	10 (12.7)
Other ethnic group	12 (11.9)	1 (4.5)	11 (13.9)
Marital Status [n (%)]			
Married	29 (28.7)	6 (27.3)	23 (29.1)
Divorced	12 (11.9)	4 (18.2)	8 (10.1)
Widowed	44 (43.6)	7 (31.8)	37 (46.8)
Single	16 (15.8)	5 (22.7)	11 (13.9)
Years in general education *M, SD* (range)	10.2, 3.73 (0–20)	10.8, 3.40 (6–20)	10.0, 3.83 (0–20)
Highest completed level of education [n (%)]			
Higher degree	1 (1.0)	1 (4.5)	0 (0)
Degree	3 (3.0)	2 (9.1)	1 (1.3)
A level (or equivalent)	3 (3.0)	0 (0)	3 (3.8)
HNC/HND (or equivalent)	3 (3.0)	0 (0)	3 (3.8)
NVQ (or equivalent)	1 (1.0)	1 (4.5)	0 (0)
GCSE (or equivalent)	7 (6.9)	2 (9.1)	5 (6.3)
No qualification	81 (80.2)	16 (72.7)	65 (82.3)
Other	2 (2.0)	0 (0)	2 (2.5)

*M* Mean, *SD* Standard deviation, *HNC/HND* Higher National Certificate/Higher National Diploma, *NVQ* National Vocational Qualification, *GCSE* General Certificate of Secondary Education

### Orofacial pain

The prevalence of orofacial pain, according to the OPS-NVI, during rest in all 101 participants, was 11.9% (95% C.I. 5.9, 18.8). Five participants were receiving parenteral nutrition, which precluded them from being observed during chewing. The prevalence of orofacial pain during chewing, in the remaining 96 participants, was 21.9% (95% C.I. 14.6, 31.3). The prevalence of orofacial pain, according to self-report is shown in Table 3. Participants who were no longer able to self-report pain, were significantly more likely to have orofacial pain, according to the OPS-NVI, during rest (*X*^2^ (1, *n* = 101) = 5.110, *p* = 0.024) and during chewing (*X*^2^ (1, *n* = 96) = 12.315, *p* < 0.001) than participants who were able to communicate the presence or absence of pain.

**Table 3:** Prevalence of orofacial pain in people with dementia in the acute hospitals

	N	Total		N	Hospital 1		N	Hospital 2	
		n (%)	95% CI of %		n (%)	95% CI of %		n (%)	95% CI of %
OPS-NVI complete cohort									
Resting	101	12 (11.9)	5.9–18.8	22	4 (18.2)	3.8–34.8	79	8 (10.1)	3.9–17.3
Chewing	96	21 (21.9)	14.6–31.3	20	6 (30.0)	11.1–52.4	76	15(19.7)	11.1–28.9
OPS-NVI in verbal P									
Resting	56	3 (5.4)	0.0–11.7	14	1 (7.1)	0.0–23.5	42	2 (4.8)	0.0–12.2
Chewing	55	5 (9.1)	1.9–16.7	13	2 (15.4)	0.0–37.5	42	3 (7.1)	0.0–16.2
OPS-NVI in non-verbal P									
Resting	45	9 (20.0)	9.1–32.6	8	3 (37.5)	0.0–75.0	37	6 (16.2)	5.6–28.1
Chewing	41	16 (39.0)	23.5–53.7	7	4 (57.1)	16.7–100.0	34	12 (35.3)	19.2–52.0
Self-report in verbal P									
Resting	56	3 (5.4)	0.0–12.7	14	1 (7.1)	0.0–23.5	42	2 (4.8)	0.0–11.8
Chewing	55	6 (10.9)	3.6–20.0	13	2 (15.4)	0.0–37.5	42	4 (9.5)	0.0–19.5

*OPS-NVI* Orofacial-Pain Scale for Non-Verbal Individuals, *CI* Confidence interval, *P* participants

### Oral health

Descriptive data of medication usage and oral health factors, of both dentate and edentate participants, are given in Table 4. Of all dentate participants, 43 participants (55.8%) had at least one retained root. Dentures were worn by 52 participants, including full dentures, frame dentures, and partial dentures. These participants included both dentate and edentate participants. Some participants used to wear dentures, but were not wearing them at the moment of assessment.

**Table 4:** Descriptive analysis of oral health factors in people with dementia in the acute hospitals

	N	Total sample	Minimum – Maximum Score
Medication [n (%)]	101		–
Analgesics		62 (61.4)	
Antidepressants		19 (18.8)	
Antiepileptics		9 (8.9)	
Antipsychotics		3 (3.0)	
Other		101 (100)	
Dental status [n (%)]	101		–
Dentate		77 (76.2)	
Edentate		24 (23.8)	
Last visit dentist [n (%)]	101		–
< 6 months ago		11 (10.9)	
6–12 months ago		14 (13.9)	
> 12 months ago		70 (69.3)	
Unknown		6 (5.9)	
Brushing [n (%)]	101		–
> 2× daily		8 (7.9)	
2× daily		18 (17.8)	
1× daily		46 (45.5)	
Never		20 (19.8)	
Something else		9 (8.9)	
Indicated swallowing quality [n (%)]	101		–
Good		56 (55.4)	
Moderate		21 (20.8)	
Bad		20 (19.8)	
Impossible		4 (4.0)	
Indicated chewing quality [n (%)]	101		–
Good		44 (43.6)	
Moderate		36 (35.6)	
Bad		21 (20.8)	
Impossible		0 (0)	
Food [n (%)]	101		–
Normal		43 (42.6)	
Consistency adjusted		58 (57.4)	
Mouth care [n (%)]	81		–
Independent		56 (69.1)	
By nurse/caregiver		12 (14.8)	
Both		13 (16.0)	
Difficulties mouth care [n (%)]	69		–
No		64 (92.8)	
Somewhat		2 (2.9)	
Yes		3 (4.3)	
Extra-oral abnormalities [n (%)]	101	33 (32.7)	–
Present teeth *M, SD* (range)	77	14.7, 7.48 (0–31)	0–32
Retained roots *M, SD* (range)	77	1.55, 2.15 (0–11)	0–28
OU *M, SD* (range)	77	2.30, 3.46 (0–14)	0–16
DI of the OHI-S *M, SD* (range)	77	2.28, 0.70 (0.5–3.0)	0.0–3.0
Upper denture [n (%)]	49		–
Full		39 (79.6)	
Frame		5 (10.2)	
Partial		5 (10.2)	
Retention upper denture [n (%)]	25		–
Good		5 (20.0)	
Moderate		9 (36.0)	
Bad		11 (44.0)	
Lower denture [n (%)]	38		–
Full		24 (63.2)	
Frame		4 (10.5)	
Partial		10 (26.3)	
Retention lower denture [n (%)]	19		–
Good		3 (15.8)	
Moderate		4 (21.1)	
Bad		12 (63.2)	
Occlusion dentures [n (%)]	26		–
Good		9 (34.6)	
Moderate		11 (42.3)	
Bad		6 (23.1%)	
Vertical dimension [n (%)]	27		–
Normal		15 (55.6)	
Open bite		0 (0)	
Deep bite		12 (44.4)	
DHI [n (%)]			–
Excellent		4 (13.8)	
Fair		9 (31.0)	
Poor		16 (55.2)	

*M* Mean, *SD* Standard deviation, *OU* Occlusal Units, *DI* Debris Index, *OHI-S* Simplified Oral Hygiene Index, *DHI* Denture Hygiene Index

### Associations between orofacial pain and oral health factors

Several oral health factors were significant predictors for the presence of orofacial pain during rest:

- Never brushing their teeth instead of once a day (OR 6.14; 95% C.I. 1.36, 27.85)
- Subjective indication of bad chewing quality (OR 10.50; 95% C.I. 1.95, 56.56)
- Having the consistency of the food adjusted (OR 9.83; 95% C.I. 1.22, 79.39)
- Having mouth care done by a nurse or other caregiver instead of being independent (OR 8.83; 95% C.I. 1.66, 46.99)
- The presence of extra-oral abnormalities (OR 33.50; 95% C.I. 4.09, 274.39)

The following oral health factors were significant predictors for orofacial pain during chewing:

- Never brushing their teeth instead of once a day (OR 3.60; 95% C.I. 1.08, 12.01)
- Subjective indication of bad chewing quality (OR 4.96; 95% C.I. 1.32, 18.74)
- Having the consistency of the food adjusted (OR 3.12; 95% C.I. 1.04, 9.37)
- Having mouth care done by a nurse or other caregiver instead of being independent (OR 4.26; 95% C.I. 1.07, 17.02)
- The presence of extra-oral abnormalities (OR 12.80; 95% C.I. 4.04, 40.53)
- The Debris Index of the OHI-S (OR 2.78; 95% C.I. 1.03, 7.54)

**Fig. 2:**
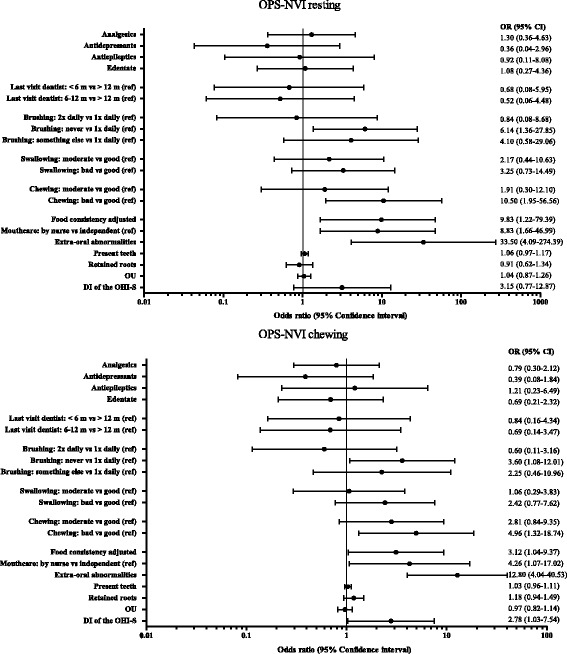
Associations of oral health factors with the presence of orofacial pain during rest (top) and chewing (bottom), according to the OPS-NVI, in all 101 participants. Odds ratios were calculated by performing binary logistic regression. OPS-NVI = Orofacial-Pain Scale in Non-Verbal Individuals, m = months, vs = versus, ref. = reference category, OU = Occlusal Units, DI = Debris

The odds ratios, with corresponding confidence intervals for all oral health factors are also shown in Fig. 2.

## Discussion

The prevalence of orofacial pain in people aged 70 years or older with dementia in the acute hospital, according to the OPS-NVI, was 11.9% during rest, and 21.9% during chewing. Participants who were no longer able to self-report pain, were significantly more likely to have orofacial pain than participants who were able to communicate about their pain. This vulnerable group of patients is where suffering may be missed. This difference may be explained by the fact that patients, who were still able to self-report pain, are able to request analgesia [21]. In this study, 61.4% of participants received analgesics on the day of the assessment. Drug usage could affect pain behaviour, and could mask the presence of pain-causing dental problems. Therefore, it is important to realize the prevalence of orofacial pain in this population could be higher than 11.9% at rest and 21.9% during chewing. The average oral hygiene of dentate participants was poor. During the data collection, participants or carers often indicated that the teeth were not brushed, due to hospitalization. Of all dentures, 55.2% had a poor hygiene. Furthermore, of all 77 dentate participants, 55.8% had at least one retained root. Indication of chewing quality, brush frequency, consistency of the food, presence of extra-oral abnormalities, person who performed mouth care, and oral hygiene in dentate participants were significant predictors for the presence of orofacial pain as rated by the OPS-NVI.

Previous studies reporting the prevalence of orofacial pain in people with dementia show a range of 7.4–20.7% [9]. Other studies confirm that older people with dementia have a higher accumulation of plaque, have a higher prevalence of caries, are more likely to have retained roots, and are in more need of dental treatment [9]. However, the use of dental treatment services is decreased in this population [22]. In the current study, 69.3% of people with dementia have not been to the dentist in the past year. In older people, hospitalization is associated with a further decrease of oral health, due to a poorer oral care [23, 24].

### Strengths and limitations

This is the first study investigating orofacial pain in people with dementia in acute hospital wards. A further strength is the inclusion of participants without capacity via the use of consultees. Without the use of consultees, there would be a risk of recruitment bias.

To identify orofacial pain in people who are unable to communicate verbally, observational tools are needed [2]. However, it is important to acknowledge the possibility of misinterpreting behaviour. For example, frowning can be interpreted as pain, but could also be caused by another cause of distress [25]. Participants were admitted to the hospital for medical reasons, which could also have caused the observed pain behaviour. The OPS-NVI is currently being validated and requires further validation to examine how well it discriminates between pain and distress.

To evaluate oral health, a brief oral assessment at the hospital site was performed. This did not enable a full dental diagnosis where a more extended oral examination is required.

The calculated sample size was not met, due to practical reasons and recruitment challenges (e.g. difficulties gaining consultee consent). Furthermore, univariate logistic regression was performed to explore associations between orofacial pain and oral health factors, without taking confounding factors into account. The presence of orofacial pain could be influenced by drugs, the type of dementia, and/or the severity of dementia. To perform logistic multivariable regression with confounding variables, a larger sample is required.

### Clinical implications

Poor oral health is common in people with dementia, and often worsened during hospital admission [14, 23]. The current findings show that the oral health of older people with dementia admitted to acute hospital wards is poor and that several oral health factors were significant predictors for the presence of orofacial pain. Poor oral health is a known risk factor for orofacial pain, and may impair general health and quality of life [7, 9, 26]. Poor dental status is also related to a higher mortality risk [27]. Moreover, approximately 10% of cases of death from pneumonia in older people could be prevented by improving oral hygiene [28]. Most oral health problems could be detected and treated by a dentist, however, people with dementia do not often visit the dentist [9]. Therefore, admission to the acute hospital could be an opportunity for oral health assessment, and dental treatment. To improve oral health care management in the acute hospital, development of guidelines and training and support for nursing staff are necessary. Studies, conducted on intensive care units and in care homes, showed improved oral health after introducing dental training programmes [29, 30].

In this study, 57.4% of participants had their consistency of food adjusted. Furthermore, the average number of OU in the dentate participants was 2.30 (SD 3.46), indicating impairment of food comminution and mastication [31]. Several studies suggest a causal relationship between mastication and cognitive abilities [32]. It is possible that improving the ability to chew, may help to stabilize, or even improve cognitive functioning and ensure quicker recovery during their acute hospital stay [32].

The OPS-NVI was used to identify orofacial pain. Until further validation has been conducted, we suggest the approach of Herr et al. to identify orofacial pain in non-verbal individuals is used in clinical situations [33]. This includes anticipating the presence of possible pain-causing conditions, establishing a baseline behaviour, and identifying pain indicators [33]. An empirical trial of simple analgesics could be used to clarify whether behavioural changes are caused by pain [33].

## Conclusions

The prevalence of orofacial pain, according to the OPS-NVI, in people aged 70 years or older with dementia in UK acute hospital wards was 11.9% at rest and 21.9% whilst chewing. The oral health status in both dentate and edentate participants admitted to acute hospitals was poor and they are more likely to develop orofacial pain. Improving oral care in acute hospital patients with dementia may significantly reduce pain and suffering in this population. The current available evidence in literature on orofacial pain in this frail population is insufficient, and has produced variable findings. This emphasizes the urgent need for further research in this area.

## Additional file

Additional file 1: Sample size calculation. The sample size calculation is clearly described in Additional file 1. (DOCX 15 kb)

## Abbreviations

CI: Confidence Interval
DHI: Denture hygiene index
DI: Debris index
FPS-R: Faces Pain Scale Revised
GCSE: General Certificate of Secondary Education
HNC/HND: Higher National Certificate/Higher National Diploma
M: Mean
NRS: Numeric rating scale
NVQ: National vocational qualification
OHI-S: Simplified oral hygiene index
OPS-NVI: Orofacial-pain scale for non-verbal individuals
OU: Occlusal units
P: Participants
SD: Standard deviation
UK: United Kingdom
VDS: Verbal descriptor scale
