# Supplementary material for: Oral health and orofacial pain in people with dementia admitted to acute hospital wards: observational cohort study
Source: BMC Geriatr. 2018 May 23;18:121. doi: 10.1186/s12877-018-0810-7 (PMC5966900; doi:10.1186/s12877-018-0810-7)
Supplement: Supplementary file 1 — Sample size calculation. The sample size calculation is clearly described in Additional file 1. (DOCX 15 kb) [file 12877_2018_810_MOESM1_ESM.docx]

**Additional file 1: Sample size calculation**

The following simple formula was used:

*n* = (Z^2^P(1-P))/*d*^2^

where *n* = sample size,

Z = Z statistic for a level of confidence

P = expected prevalence or proportion (in proportion of one)

*d* = precision

For the level of confidence of 95%, Z value is 1.96.

With an assumed prevalence of 12%, P is 0.12.

With a precision of +/-5 percentage points (0.05), *d* should be set at 0.05.

*n* = (1.96^2^ x 0.12 x (1-0.12))/0.05^2^ = 162
